# Soil bacterial endemism and potential functional redundancy in natural broadleaf forest along a latitudinal gradient

**DOI:** 10.1038/srep28819

**Published:** 2016-06-30

**Authors:** Yuguang Zhang, Jing Cong, Hui Lu, Ye Deng, Xiao Liu, Jizhong Zhou, Diqiang Li

**Affiliations:** 1 Institute of Forestry Ecology, Environment and Protection, and the Key Laboratory of Forest Ecology and Environment of State Forestry Administration, Chinese Academy of Forestry, Beijing 100091, China; 2 School of Minerals Processing and Bioengineering, Central South University, Changsha, 410083, China; 3 College of Life and Environment Sciences, Minzu University of China, Beijing 100081, China; 4 Research Center for Eco-Environmental Science, Chinese Academy of Sciences, Beijing 100085, China; 5 Institute for Environmental Genomics, Department of Botany and Microbiology, University of Oklahoma, Norman OK 73019, USA.

## Abstract

Microorganisms play key roles in ecosystem processes and biogeochemical cycling, however, the relationship between soil microbial taxa diversity and their function in natural ecosystems is largely unknown. To determine how soil bacteria community and function are linked from the local to regional scale, we studied soil bacteria community composition, potential function and environmental conditions in natural and mature broadleaf forests along a latitudinal gradient in China, using the Illumina 16S rRNA sequencing and GeoChip technologies. The results showed strong biogeographic endemism pattern in soil bacteria were existed, and the spatial distance and climatic variables were the key controlling factors for this pattern. Therefore, dispersal limitation and environmental selection may represent two key processes in generating and maintaining the soil bacterial biogeographic pattern. By contrast, the soil bacterial potential function is highly convergent along the latitudinal gradient and there were highly differing bacterial community compositions, and the soil chemistry may include the main factors active in shaping the soil bacterial potential function. Therefore, the soil bacterial potential function may be affected by local gradients in resource availability, and predicting soil bacterial potential function requires knowledge of abiotic and biotic environmental factors.

The biological community structure and function in ecological processes are intimately linked, and their relationship is one of the central issues in ecology theory^1^,^2^. Comparative studies have revealed that the taxa diversity and structure for plants and animals often alter ecosystem properties, such as primary productivity^3^, decomposition rates^1^, resistance and resilience to perturbations^4^, and nutrient cycling^1^,^5^. Microorganisms are one of the most abundant and diverse organism types and play key roles in ecosystem processes and biogeochemical cycling of carbon, nitrogen, sulphur, phosphorus and metals, and biodegradation or stabilisation of environmental contaminants^6^,^7^. Therefore, identifying and understanding microbial community structure, function and their relationships are particularly important, and understanding the extent of structure-function relationships between microorganisms over large spatial scales is a major goal of ecological research^2^. However, the relationship between soil microbial diversity and microbial function is largely unknown^8^.

Because of the high diversity, complexity and plasticity of environmental microorganisms, and the limitations of study methods, it is very difficult to understand the detailed ecosystem processes facilitated by microorganisms. Therefore, our understanding of the microbial distribution and functional traits in nature is currently limited^7^. With the development of DNA technologies, our understanding of the phylogenetic and taxonomic structure of soil microbial communities continues to expand, and recent work has documented how soil bacterial communities are affected by specific environmental changes or disturbances^7^,^9^,^10^. Some current research has reported the relationship between soil microbial community structure and function, but the results of such studies were inconsistent or contradictory, including functional redundancy^2^ or strong positive correlations^11^, which might be because: 1) the assessment methods may not fully reveal the real microbial function, such as the enzyme activity or particular functional gene^2^,^11^; 2) only the overall correlation between microbial taxonomic composition and the functional attributes is included, while excluding distinct taxa with specific functional attributes^11^,^12^; 3) certain microbial ecological processes are only apparent or important at a particular scale^10^,^13^,^14^, and therefore a comprehensive understanding of community-environment-function interactions is required at multiple scales^2^. Therefore, it is difficult to predict the functional attributes or diversity based on the biogeographical patterns in the taxonomic or phylogenetic structure of soil microbial communities^7^.

Microbial ribosomal gene copy number has proved to have the potential to impact ecosystem function and predict the response of ecosystems to environmental change^7^,^15^. In DNA hybridisations, signal intensity is correlated with gene copy number and abundance of the organisms carrying these genes^16^. GeoChip contains probes corresponding with the genes encoding key enzymes involved in various biogeochemical cycling and has become a powerful and high-throughput tool for the analysis of microbial communities and ecosystem functions^17^,^18^,^19^. A significant correlation between cellulose gene signal intensity and cellulose activity in the soil, and the notable (*P* < 0.1) correlations between dehydrogenase gene signal intensities and activity, urease gene signal intensities and soil ammonium and sulphite reduction gene signal intensities and soil sulphur levels, also points to a relationship between gene copy number and soil function^17^. Yergeau *et al*. also showed a significant correlation between cellulase enzyme activity and the number of cellulase gene variants detected using GeoChip^20^. All these results indicated that GeoChip can be used not only to analyse the structure, functional activity and dynamics of microbial communities, but also to link microbial communities with ecosystem processes and functions^21^.

Forests are one of the most important and complex biomes in the terrestrial ecosystem. They not only contain most of all known plant and animal species, but also provide a variety of key resources and ecosystem services to humans, including food, drinking water, timber and medicines. Soil microbial communities are expected to be particularly complex under forest ecosystems and a deep analysis of soil microbial communities and their roles in ecological processes would improve our understanding of biogeochemical elemental cycles^10^. To determine how soil bacteria community structure and function in the natural forest ecosystem are linked from the local to regional scale, we studied soil bacterial species composition, potential function and environmental conditions in 240 soil samples taken from 24 national natural reserves along a latitudinal gradient in China (Fig. S1 and Table S1).

## Results

### The soil bacterial community structure and geographic endemism

The soil bacterial community structure was detected by 16S rRNA Illumina sequencing. A total of 11,444,052 quality 16S rRNA sequences were detected in all study sites, with 26,026–93,786 sequences per sample plot. Phylogenetic analysis showed that at least 29 known bacterial phyla were presented in these forest sites and all of these 29 detected phyla were presented at all sample sites. The relatively dominant phyla were *Acidobacteria*, *Actinobacteria*, *Proteobacteria* and *Verrucomicrobia*, and these four phyla accounted for over 75% of the bacterial sequences (Table S2). The lowest and highest bacterial Shannon-Weaver index at the 24 sampling national nature reserves was 6.96 and 8.40, respectively.

**Figure 1:**
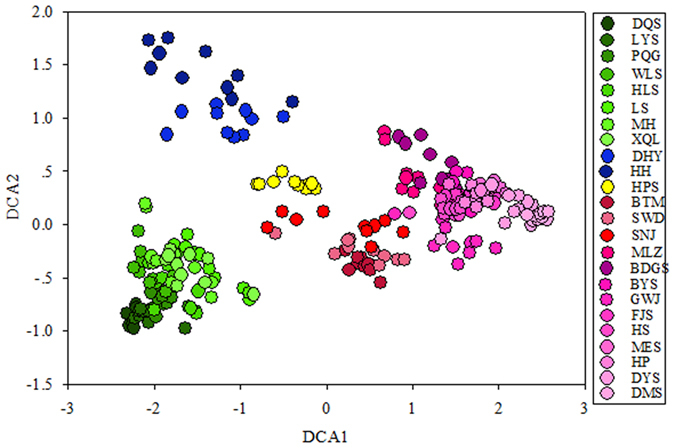
Detrended correspondence analysis (DCA) of soil microbial community based on high-throughput 16S rRNA sequences. The DCA was analyzed based on the relative abundances of OTUs. Points represent individual sample collected from each sampling location at each plot in each field site (n = 240).

Detrended correspondence analysis showed that bacterial community structure has distinct geographic endemism features among samples (Fig. 1). In Fig. 1, the bacterial community was divided into four clusters (different colour sites) along the latitudinal gradient in the natural and mature broadleaf forest soil. The green sites were from DQS (E 111.25°, N 40.83°) to XQL (E 110.50°, N 34.43°) along the latitudinal gradients, the blue sites were DHY (E 110.01°, N 31.53°) and HH (E 111.55°, N 30.08°), the yellow site is HPS (E 110.53°, N 30.04°), and the red sites were BTM (E 111.94°, N 33.49°), SWD (E 110.75°, N 32.43°), SNJ (E 110.36°, N 31.49°), and from MLZ (E 110.22°, N 30.06°) to DMS (E 108.44°, N 23.49°).

**Figure 2:**
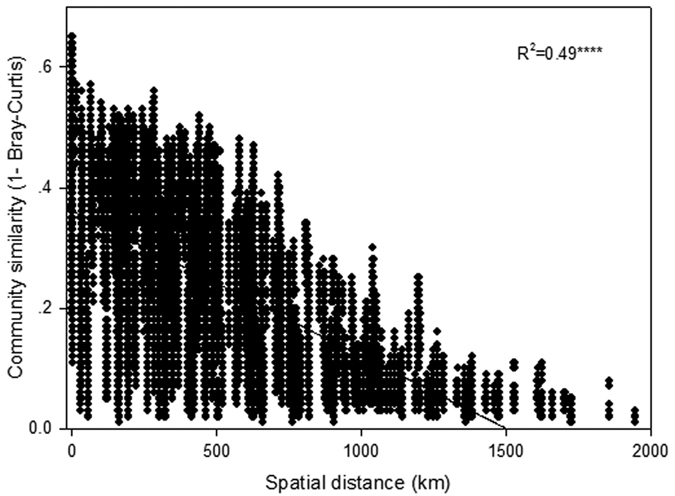
The regression relationship between the soil bacterial Bray-Curtis index and sampling sites change in spatial distance. Asterisks represent significance of correlation (****P < 0.0001).

The decay of soil microbial community similarity with geographical distance was analysed by the bacterial 16S rRNA Bray-Curtis index and a significant distance - decay relationship (*P* < 0.001) was found (Fig. 2). Figure 2 showed that the spatial distance explained about 49% of the total variation of bacterial community composition. The z value of the taxa -area relationship for all the sequences and *Acidobacteria*, *Actinobacteria*, *Proteobacteria* and *Verrucomicrobia* phyla showed that there was a steep relationship (Table 1). According to Table 1, the z value was 0.115 for all the sequenced 16S rRNA, the *Acidobacteria* phylum had the highest z value (0.139) of the dominant phyla, and the lowest z value (0.097) was found in the *Protecobacteria* phylum. Thus, there was a significant distance–decay relationship in the structure of soil bacterial communities along the latitudinal gradient in the natural mature broadleaf forest soil.

### The soil bacterial potential function

To analyse the soil bacterial potential function, 18 key bacterial functional gene categories involved in carbon, nitrogen and phosphorus cycles were selected and analysed. The soil bacterial key functional gene categories were highly correlated (Table S3), thus, to reduce the measured functional gene categories to a reasonable number of predictors, we used principal component analysis on the log-transformed variables. After examining screen plots, we chose to retain the first two principal components, which explained 40.9% (PC1 = 28.1% and PC2 = 12.8%) of the variation in soil bacterial functional gene categories. The first principal component was associated with variation in formyltetrahydrofolate synthetase (Fthfs), endoglucanase, mannanase, xylanse, phenol oxidase, phytase and polyphosphate kinase (ppk), and the second was associated with fructose-1, 6-bisphosphatase (FBPase), cellobiase, alpha amylase (amyA), nitrite reductase (nirS/nirK) and nitrogenase reductase (nifH) (Table S3).

**Figure 3:**
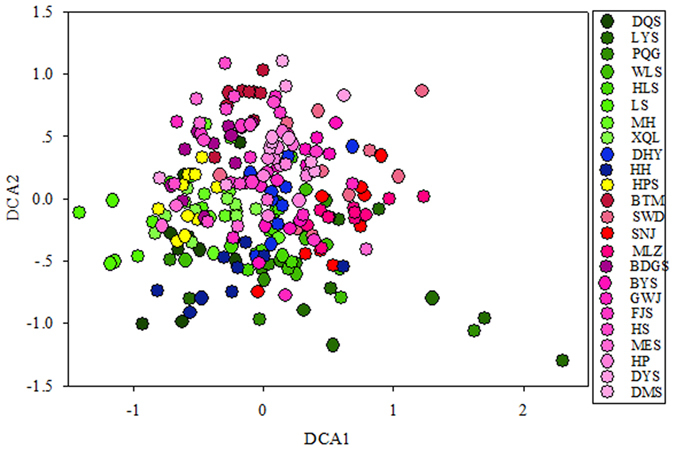
Detrended correspondence analysis (DCA) for soil microbial 18 functional gene families for sum signal intensity. The 18 functional gene families were formyltetrahydrofolate synthetase (FTHFS), fructose-1, 6 – bisphosphatase (FBPase) and ribulose-1, 5- bisposphate carboxylase/oxygenase (Rubiso) for carbon fixation, cellobiase, endoglucanase, chitinase, mannanse, xylanase, phenol oxidase and alpha amylase for carbon degradation, encoding urease (ureC), nitrate reductase (narG), nitrite reductase (nirS/K), nitrous oxide reductase (nosZ), and nitrogenase reductase (nifH) for nitrogen cycling, phytase, exopolyphosphatase (ppx) and polyphosphate kinase (ppk) for phosphorus cycling. Points represent individual sample collected from each sampling location at each plot in each field site (n = 240).

In contrast to bacterial communities, the soil bacterial key functional gene categories did not differ along the latitudinal gradient (Fig. 3). In Fig. 3, the soil bacterial key functional gene categories show no significant geographic clustering in the broadleaf forest along the latitudinal gradient; therefore, the soil bacterial potential functions had no significant geographic features and maybe highly functional redundancy.

### The effect of environmental variables on bacterial diversity and potential function

The Pearson relationship analysis between measured variables showed that soil chemical variables and plant diversity were generally highly correlated (Table S4). For example, the soil moisture, total soil organic carbon, total soil nitrogen, nitrate nitrogen and available nitrogen were highly correlated. However, soil pH was weakly correlated with soil phosphorus and nitrate nitrogen. The plant diversity was highly correlated with the soil moisture, soil organic carbon, available nitrogen and nitrate nitrogen. To decrease the self-correlation, we performed principal component analysis on the soil chemical variables (Fig. S2). After examining screen plots, we chose to retain the first two principal components, which explained 66.9% (PC1 = 51.1% and PC2 = 15.8%) of the variation in soil chemical variables. The first principal component was associated with variation in soil moisture, total organic carbon and total nitrogen, and the second was associated with soil pH and total phosphorus. Climatic variables were also highly correlated and separated into three principal components that explained 95.0% (PC1 = 78.6%, PC2 = 9.3% and PC3 = 7.2%) of the variation in climate across sites (Dataset S1). The first principal component was associated with variation in annual mean temperature and annual precipitation.

To determine the factors that influence soil bacterial community composition along the latitudinal gradient in the broadleaf forests, we conducted multiple regression analysis with spatial distance, soil chemistry PC1 and PC2, climate PC1, PC2, PC3, elevation and plant diversity (Shannon-Weaver index) as independent variables and with soil bacterial Bray–Curtis community dissimilarity between sites as the dependent variable. The analysis showed that the spatial distance (R^2^ = 0.49) and climate factor PC1 (R^2^ = 0.51) had a significant impact on the soil bacterial community structure (Table 2). The soil chemistry, elevation and plant diversity also had significant effects on soil bacterial community variation (Table 2).

The univariate analyses were conducted to determine the effect of environmental factors on soil bacterial potential function. Multiple regression analysis showed there was a weak relationship between spatial distance and the bacterial key functional gene categories (0.002 for PC1 and 0.04 for PC2) (Table 2, Dataset 2). Therefore, the spatial distance and bacterial community structure had a limited effect on the bacterial potential function. Multiple regression analysis showed that the soil chemistry had a strong relationship (*P* = 0.001) with the bacterial key functional gene categories (0.15 for PC1 and 0.19 for PC2) (Table 2, Dataset 2). Therefore, the soil chemistry may include the key factors in shaping the soil bacterial potential function along the latitudinal gradient in natural and mature broadleaf forest. According to Table 2, the elevation (0.22), climate factors (0.16 for PC2) and plant diversity (0.11) had significant relationships with the soil bacterial potential function (PC2).

## Discussion

In recent years, increasing numbers of researchers have provided considerable evidence that microorganisms display significant biogeographic patterns, similar to plants and animals. The decay of community similarity with geographic distance relationship is fundamental to our understanding of the biogeographic patterns of global biodiversity and its z value is a measure of the rate of turnover of species across space. The – (regression coefficient)/2 of a linear regression has been used to estimate the z value of the distance-decay relationship^22^ at local^23^, regional^24^, and global^9^ scales. In this study, the experimental design and method is consistent with typical biogeographical studies^23^ and we believe that the z values could be representative of the biogeographic patterns of soil microbial communities in forest soils. In our study, the z value (0.115) was higher than others previously observed for microorganisms in the marine^25^, tropical lake sediment^26^, salt marsh^23^, and forest environments^9^,^27^. This result suggested that the soil bacteria in the temperate and subtropical forest soil may have a higher turnover rate at these spatial and taxonomic scales. In previous research, very few studies have reported the z values in forest soils and z values ranged from 0.003 at the global scale using terminal restriction fragment length polymorphism (T-RFLP)^9^, 0.0626 at the local scale using GeoChip^27^ to 0.42 at the local scale using T-RFLP^28^. These z values were significantly different, which may be caused by the following: 1) the z value may be underestimated for the taxonomic resolution in T-RFLP and for the interested genes exhibit high spatial variability in GeoChip^27^, and 2) the spatial scale used for sampling at the local scale may not be suitable for describing the influences of environmental heterogeneity and geographic distance on microbial community diversity patterns^27^. Therefore, it is difficult to directly compare z values from different methods and at different spatial scales.

Many researchers also have begun to suggest some theoretical frameworks to evaluate and explain the processes that generate and maintain the microbial biogeographic patterns^29^. Selection, drift, dispersal and mutation are four such processes and the challenge is now in identification of the relative importance of each of the four processes^29^. The multiple regression analysis showed that the spatial distance (R^2^ = 0.49) and climatic variables (temperature and precipitation) (R^2^ = 0.51) were the key factors in shaping the soil bacterial biogeographic patterns in the forest soils. The temperature and precipitation were significantly different along the latitudinal gradient from the temperature zone to the subtropical zone in this study. Therefore, the dispersal limitation and environmental selection may be the two key processes in generating and maintaining the soil bacterial biogeographic pattern. The literature also suggests that both environmental selection and historical processes (including dispersal limitation) are key in shaping the microbial distribution at different spatial scales, habitat types and taxonomic resolutions^2^,^9^,^29^. However, most of the studies found that environmental selection seems to have a stronger influence than historical processes^29^,^30^. Most of the studies (68%) also reported that spatial distance provided evidence that historical processes, including dispersal limitation, influence microbial composition^23^,^25^,^29^. For example, Talbot *et al*. showed that the soil fungal communities are most consistent with a strong role for dispersal limitation as a driver of community turnover^2^.

A comprehensive understanding of microbial systems requires understanding of community–environment–function interactions at multiple scales^2^. At present, we know little regarding the relationship between microbial community composition and distribution of microbial functional traits in nature^7^. Recently, studies that have explicitly considered the composition and diversity of microbial communities at the local scale have also observed relationships between bacterial community composition and biochemical function in soils^10^,^14^. However, it is unclear whether a link between bacterial community structure and function for microbial communities operates on a larger scale^2^. Knowing the structure of regional taxa will be important for understanding the microbial function over large geographical regions^2^. Soil biochemical properties and enzyme activity are important indices for detecting the microbial function; however, it is difficult to identify the relationship between distinct taxa and specific functional attributes^12^. In this study, the functional gene categories acted as the bacterial potential functions and thus we can assume the relationship between distinct microbial taxa and specific function. To our knowledge, this study is the first attempt to detect the relationship between soil bacterial community composition and function at large scales, and the results showed there was clear soil bacterial functional redundancy in the natural and mature forests along the latitudinal gradient. These results may be explained in two ways. The first is that the soils in the natural and mature broadleaf forests may have similar functions for the carbon, nitrogen and phosphorus cycle. The second is that microorganisms are assumed to evolve rapidly and closely related taxa may have very different physiologies and environmental tolerances^12^. Therefore, microbial taxa distributions have been assumed to be of little value for predicting functional attributes.

In this study, the soil bacterial potential function appeared to have a strong relationship with soil chemistry (R^2^ = 0.14 and R^2^ = 0.19). The spatial distance and bacterial community structure had a weak relationship with the soil bacterial potential function. Therefore, the total explained variation was low for the bacterial potential function and the factors controlling the soil bacterial function in the forest soil could be very complicated^27^. The possible explanations for these results may be associated with some key controlling factors that were not measured, such as the bacterial competitions and interactions, and levels of soil aggregates and the labile carbon pool. Therefore, the soil bacterial potential function is affected by local gradients in resource availability, and predicting bacterial potential function requires knowledge of local biotic and abiotic environmental conditions.

## Materials and Methods

### Soil sampling

To decrease the confounding effects of vegetation type that could drive cross-biome differences in bacterial community structure-function relationships, we established sampling sites in natural and mature broadleaf forest types along a latitudinal gradient from north (N 40°, E 111°) to south (N 23°, E 108°) in national natural reserves in China (Fig S1 and Table S1). Sampling was carried out in July to October, 2012. At each site, ten plots (20 × 20 m) were selected with about 20 m between adjacent plots. The GPS locations were recorded using a GPS receiver. Detailed information on the sampling sites is presented in Table S1. Ten to fifteen soil cores (0–10 cm depth) distance over 1 meter from tree trunk were taken from each plot and combined to obtain about 400 g of soil. Samples were sieved through 2-mm mesh to remove roots and stones, then mixed thoroughly. About 100 g of each bulked soil sample was preserved at −80 °C for DNA extraction and the remaining soil was kept at room temperature for soil chemistry characteristic analysis.

### Plant diversity and soil chemistry properties analysis

Plant properties were surveyed in each plot, including the plant species, number, height and canopy of each tree and shrub, and diameter at breast (1.3 m) height of trees (DBH > 5cm) and shrubs (DBH 1–5 cm). Soil moisture, soil pH, total soil organic carbon (SOC) and total nitrogen (TN), available nitrogen (AN), nitrate nitrogen (NN), ammonium nitrogen (AMN), total phosphorous (TP) and rapid available phosphorous (RAP) were measured as previously described^31^.

### Climate data

The climate data for each sampling plot was obtained by using the WorldClim global climate dataset (from about 1950 to 2000) based on the recorded GPS location information of the latitude and longitude^32^. We chose to use the Bioclim variables, which summarise monthly precipitation and temperature into 19 meaningful biological variables^2^, such as the annual mean temperature and annual precipitation (Dataset S1). To reduce the effect of data that did not conform to assumptions of normality and homogeneity of variance, values were log-transformed before analysis.

### Soil microbial DNA extraction

Soil microbial DNA in each sampling plot was extracted by freeze-grinding mechanical lysis and purified twice using low melting point agarose gel followed by phenol-chloroform-butanol extraction^33^. The purified DNA quality was assessed by the ratios of 260:280 nm and 260:230 nm. Final DNA concentrations were quantified with a PicoGreen method using a FLUO star Optima (BMG Labtech, Jena, Germany).

### Bacterial community composition and structure

To determine the bacterial community composition in each soil sample, we used Illumina high-throughput sequencing of bacterial DNA amplicons from each soil sampling plot. Based on the V4–V5 hypervariable regions of bacterial 16S rRNAs, the PCR primers, F515: GTGCCAGCMGCCGCGGTAA, and R806: GGACTACHVGGGTWTCTTA were selected, and the primers combined with adapter sequences and barcode sequences^34^,^35^. The PCR reaction, amplification conditions, quantification and denaturation followed Ding *et al*.^36^. The sequences were run on the Miseq sequencer (Illumina, San Diego, CA, USA) for 2 × 250 bp paired-end sequencing.

Only the first 250 bp after the proximal PCR primer of each sequence was used to further analysis to minimise the effects of random sequencing errors. Sequence quality trimming was performed using Btrim^37^. Paired-end sequences were merged into full-length sequences by FLASH v1.2.5^38^. The sequences were removed if the sequences did not perfectly match the PCR primer, if the sequences had non-assigned tags, or if the read length was less than 250 bp. All sequences were aligned using the RDP Infernal Aligner (Ribosomal Database Project, Michigan State University, East Lansing, MI, USA), and the complete linkage clustering method was used to define operational taxonomic units (OTUs) using 97% identity as a cutoff^39^. The number of detected OTUs and sequences at different levels of classification were counted. Details of amplicon preparations, sequencing and data analysis (e.g., classification, OTU identification) are described in He *et al*.^21^ and Deng *et al*.^39^. The singletons were removed for downstream analyses. To standardise samples, a sub-sample of 20,000 sequences per soil sampling plot was used to compare the relative difference between samples.

### The soil bacterial potential function

The soil bacterial potential function in each sampling plot was assessed using the detected functional gene category signal intensity. GeoChip 5.0, contains >57,000 oligonucleotide probes targeting >140,000 genes in 393 gene categories involved in biogeochemical cycling of carbon, nitrogen, phosphorus and sulphur. The GeoChip 5.0 was thus used to analyse the bacterial functional gene signal intensity. The detailed information is presented on the website (http://ieg.ou.edu/). The key enzyme gene categories related to carbon, nitrogen, phosphorus and sulphur cycling were selected, including formyltetrahydrofolate synthetase (FTHFS), fructose-1, 6-bisphosphatase (FBPase) and ribulose- 1, 5-bisphosphate carboxylase/oxygenase (Rubiso) for carbon fixation, cellobiase, endoglucanase, chitinase, mannanse, xylanase, phenol oxidase and alpha amylase for carbon degradation, encoding urease (ureC), nitrate reductase (narG), nitrite reductase (nirS/K), nitrous oxide reductase (nosZ) and nitrogenase reductase (nifH) for nitrogen cycling, phytase, exopolyphosphatase (ppx) and polyphosphate kinase (ppk) for phosphorus cycling.

To produce consistent hybridisations from all samples, a whole community genome amplification was used to generate approximately 3.0 μg of DNA with 50 ng purified DNA using the TempliPhi Kit (GE Healthcare, Piscataway, NJ, USA) following the manufacturer’s instructions. Amplified DNA was labeled with a Cy5 fluorescent dye (GE Healthcare) using a random priming method^19^. All hybridisations were carried out at 45 °C for 10 h with 50% formamide using a TECAN HS4800 and arrays were scanned using the ScanArray 5000 analysis system (Perkin-Elmer, Wellesley, MA, USA). Signal intensities of each spot were measured with ImaGene 6.0 (Biodiscovery Inc., EI Segundo, CA, USA) and only the spots automatically scored as positive in the output of raw data were used for downstream data analysis.

The GeoChip data were further analysed using the following steps: (i) removing genes detected in fewer than 6 of the 10 samples from the same national natural reserve; (ii) normalizing the signal intensity of each spot by dividing the mean value of each sample of total signal intensity; and (iii) summing the total signal intensity of each functional gene category.

### Data statistical analysis

The principal components analysis was used to determine the role of different spatial and environmental factors in determining bacterial structure and function. Bacterial diversity and community structure were calculated using the Shannon-Weaver Index and relative abundance based on Illumina-sequencing. Detrended correspondence analysis was used to determine the changes in overall bacterial community structure along the latitudinal gradient in the natural broadleaf forest. The beta-diversity was calculated by using the Bray–Curtis Index. The bacterial potential function was analysed using detrended correspondence analysis, based on log transformed of the sum signal intensity of the detected functional gene category.

To determine the influencing factors in bacterial community structure, we used the multiple regressions analysis using permutation tests of significance for regression coefficients and R-suqared by the ecodist R package^40^. Multiple regression analysis was conducted with spatial distance, elevation, soil chemistry PC1 and PC2 (from principal components analysis), plant diversity or climate principal component axes as independent variables and soil bacterial Bray-Curtis community dissimilarity among soil samples (permutations of 9,999 times). We estimated the power - law exponent z with a distance-decay approach for all the bacterial sequences and the four most dominant phyla (*Acidobacteira, Proteobacteria, Actinobacteria* and *Verrucomicrobia*)^23^. To determine the effect of bacterial community, resource availability and climate on soil bacterial potential function, we conducted multiple regression analyses with spatial distance, bacterial community dissimilarity, and single regression analysis with plant diversity, elevation, soil chemistry PC1 and PC2, climate PC1 and PC2 as independent variables and soil bacterial potential function PC1 and PC2 as dependent variables (permutations of 9,999 times).

## Additional Information

**Accession codes:** Microarray data has been deposited to GEO databases and the accession number is GSE69171 (http://www.ncbi.nlm.nih.gov/geo/query/acc.cgi?acc=GSE69171). 16S rRNA sequences have been deposited to GenBank databases and the accession number is SRP062748 (http://trace.ncbi.nlm.nih.gov/Traces/sra/?study=SRP062748).

**How to cite this article**: Zhang, Y. *et al*. Soil bacterial endemism and potential functional redundancy in natural broadleaf forest along a latitudinal gradient. *Sci. Rep.* **6**, 28819; doi: 10.1038/srep28819 (2016).

## Supplementary Material

Supplementary Information

Supplementary Data 1

Supplementary Data 2

## Figures and Tables

**Table 1:** The z-values for all the sequences and the dominant phylum.

OTU Phylum	z-value	Regression coefficient	t	*P*
All sequence	0.115	−0.473	−1825.25	<0.001
*Acidobacteria*	0.139	−0.454	−1747.74	<0.001
*Proteobacteria*	0.097	−0.458	−1706.35	<0.001
*Verrucomicrobia*	0.107	−0.473	−1805.26	<0.001
*Actinobacteria*	0.133	−0.478	−1720.10	<0.001

The z values shown were determined using the distance decay approach. t and *P* values are from one-sample tests on bootstrapping (9, 999 times) for testing significance of z values.

**Table 2:** Statistics predicting bacterial community composition and soil bacterial functional gene categories.

Environmental Factor	Bray-Curtis community dissimilarity	Soil bacterial potential function PC1		Soil bacterial potential function PC2	
		R^2^	F statistic	R^2^	F statistic
Spatial distance	0.49****	0.002	85.607*	0.04	1128.58****
Elevation	0.11****	0.012	1.394	0.22	33.955****
Climate PC1	0.51****	0.002	0.207	0.16	22.625****
Climate PC2	0.001***	0.03	3.251*	0.05	6.579***
Climate PC3	0.02****	0.03	3.317*	0.08	9.975****
Soil chemistry PC1	0.08****	0.03	3.922**	0.14	18.433****
Soil Chemistry PC2	0.22****	0.15	20.934****	0.19	27.397****
Plant diversity	0.14****	0.02	2.337	0.11	15.265****
Bray-Curtis community dissimilarity		0.001	18.67*	0.05	1625.92***

Single factor statistics are generated from single regression analyses (for elevation, climate, soil chemistry, and plant diversity) or multiple regression analysis (for bacterial Bray-Curtis community dissimilarity and spatial distance). Asterisks represent significance of regression (****P < 0.0001, ***P < 0.001, **P < 0.01, *P < 0.05). PC, principal component.
