# Supplementary material for: Soil bacterial endemism and potential functional redundancy in natural broadleaf forest along a latitudinal gradient
Source: Sci Rep. 2016 Jun 30;6:28819. doi: 10.1038/srep28819 (PMC4928066; doi:10.1038/srep28819)
Supplement: Supplementary Information [file srep28819-s1.doc]

Soil bacterial endemism and potential functional redundancy in natural broadleaf forest along a latitudinal gradient

Yuguang Zhang ^1*^, Jing Cong^1, 2^, Hui Lu^3^, Ye Deng ^4^, Xiao Liu^1^, Jizhong Zhou ^5^, Diqiang Li^1^

^1^Institute of Forestry Ecology, Environment and Protection, and the Key Laboratory of Forest Ecology and Environment of State Forestry Administration, Chinese Academy of Forestry, Beijing 100091, China

^2^ School of Minerals Processing and Bioengineering, Central South University, Changsha, 410083, China

^3^ College of Life and Environment Sciences, Minzu University of China, Beijing 100081, China

^4^ Research Center for Eco-Environmental Science, Chinese Academy of Sciences, Beijing 100085, China

^5^Institute for Environmental Genomics and Department of Botany and Microbiology, University of Oklahoma, Norman OK 73019

Table S1 Soil sampling sites characteristics in 24 national nature reserves

| Site ID | Location | East longitude | North Latitude | Elevation  (m) | Forest Types | Dominant plant species | Plant Shannon-  Weaver index (SE) | Plant Pielou index (SE) | Plant species  richness |
| --- | --- | --- | --- | --- | --- | --- | --- | --- | --- |
| DQS | Daqingshan national nature reserve | 111.2472 | 40.8321 | 1677 | Temperate forest | *Quercus wutaishanica* Mayr. | 1.64±0.21 | 0.79±0.09 | 8.1 |
| LYS | Luyashan National nature reserve | 111.9721 | 38.7106 | 1780 | Temperate forest | *Quercus wutaishanica* Mayr. | 1.63±0.18 | 0.75±0.07 | 9.0 |
| PQG | Pangquangou national nature reserve | 111.4684 | 37.8596 | 1888 | Temperate forest | *Quercus wutaishanica* Mayr. | 1.63±0.16 | 0.71±0.05 | 10.1 |
| WLS | Wulushan national nature reserve | 111.1500 | 36.5579 | 1841 | Temperate forest | *Quercus wutaishanica* Mayr. | 1.46±0.33 | 0.65±0.08 | 9.2 |
| HLS | Huanglongshan national nature reserve | 110.0397 | 35.7001 | 1544 | Temperate forest | *Quercus wutaishanica* Mayr. | 2.11±0.22 | 0.75±0.06 | 16.8 |
| LS | Lishan national nature reserve | 112.0231 | 35.4239 | 1605 | Temperate forest | *Quercus wutaishanica* Mayr. | 2.06±0.23 | 0.74±0.05 | 16.4 |
| MH | Manghe national nature reserve | 112.3767 | 35.2570 | 1403 | Temperate forest | *Cyclobalanopsis glauca* (Thunb.) Oerst. | 2.14±0.24 | 0.70±0.06 | 17.6 |
| XQL | Xiaoqinglin national nature reserve | 110.5024 | 34.4320 | 1771 | Temperate forest | *Quercus mongolica* Fisch. ex Ledeb. | 2.38±0.25 | 0.78±0.05 | 21.1 |
| BTM | Baotianman national nature reserve | 111.9372 | 33.4949 | 1402 | Temperate forest | *Quercus chenii* Nakai | 1.78±0.15 | 0.83±0.07 | 8.6 |
| SWD | Saiwudang national nature reserve | 110.7511 | 32.4309 | 1108 | Subtropical forest | *Quercus serrata* Thunb. | 2.74±0.35 | 0.79±0.06 | 32.6 |
| DHY | Duheyuan national nature reserve | 110.0135 | 31.5267 | 1798 | Subtropical forest | *Populus lasiocarpa* Oliv. | 2.61±0.31 | 0.84±0.05 | 23.0 |
| SNJ | Shennongjia national nature reserve | 110.3601 | 31.4898 | 1785 | Subtropical forest | *Quercus aliena* Blume var. *acutiserrata* Maxim. ex Wenz. | 2.14±0.22 | 0.64±0.06 | 28.5 |
| HH | Houhe national nature reserve | 110.5496 | 30.0785 | 1568 | Subtropical forest | *Cyclobalanopsis glauca* (Thunb.) Oerst. | 2.23±0.20 | 0.84±0.07 | 14.5 |
| MLZ | Mulinzi national nature reserve | 110.2241 | 30.0601 | 1466 | Subtropical forest | *Cyclobalanopsis glauca* (Thunb.) Oerst. | 2.95±0.07 | 0.83±0.03 | 35.4 |
| HPS | Hupingshan national nature reserve | 110.5305 | 30.0444 | 1823 | Subtropical forest | *Populus lasiocarpa* Oliv. | 1.95±0.43 | 0.78±0.13 | 12.3 |
| BDGS | Badagongshan national nature reserve | 110.0742 | 29.7734 | 1453 | Subtropical forest | *Fagus lucida* Rehder et E. H. Wilson. | 3.12±0.16 | 0.82±0.04 | 44.5 |
| BYS | Baiyunshan national nature reserve | 109.3222 | 28.6760 | 1068 | Subtropical forest | *Schima argentea* E. Pritz. | 3.23±0.43 | 0.78±0.13 | 63.0 |
| GWJ | Gaowangjie national nature reserve | 110.0754 | 28.6629 | 927 | Subtropical forest | *Castanopsis eyrei* (Champ. ex Benth.) Tutcher. | 2.39±0.48 | 0.89±0.06 | 15.9 |
| FJS | Fanjingshan national nature reserve | 108.7372 | 27.8776 | 1046 | Subtropical forest | *Castanopsis fargesii* Franch. | 3.08±0.23 | 0.84±0.04 | 39.8 |
| HS | Huangsang national nature reserve | 110.0790 | 26.3939 | 1055 | Subtropical forest | *Carpinus chuniana* Hu. | 3.10±0.17 | 0.85±0.03 | 39.2 |
| MES | Maoershan national nature reserve | 110.4732 | 25.9080 | 1248 | Subtropical forest | *Daphniphyllum macropodum* Miq. | 2.49±0.30 | 0.73±0.07 | 30.5 |
| HP | Huaping national nature reserve | 109.9403 | 25.5622 | 1211 | Subtropical forest | *Schima argentea* E. Pritz. | 2.84±0.29 | 0.79±0.07 | 35.8 |
| DYS | Dayaoshan national nature reserve | 110.2464 | 24.1632 | 1514 | Subtropical forest | *Cyclobalanopsis sessilifolia* (Blume) Schottky. | 2.34±0.15 | 0.66±0.05 | 35.1 |
| DMS | Damingshan national nature reserve | 108.4420 | 23.4925 | 1248 | Subtropical forest | *Castanopsis eyrei* (Champ. ex Benth.) Tutcher. | 2.38±0.23 | 0.70±0.04 | 31.5 |

SE, Standard Error.

Table S2 Distribution of the dominant bacteria phylum across sites

| Site ID | Shannon-Weaver index | Acidobacteria | Actinobaceria | Armatimonadetes | Bacteroidetes | Chloroflexi | Firmicutes | Gemmatimonadetes | Planctomycetes | Proteobacteria | Verrucomicrobia |
| --- | --- | --- | --- | --- | --- | --- | --- | --- | --- | --- | --- |
| DQS | 8.26(0.02) | 27.64(0.70) | 9.19(0.83) | 0.26(0.03) | 8.26(0.29) | 0.66(0.03) | 0.90(0.08) | 2.18(0.13) | 1.46(0.07) | 33.97(0.53) | 7.39(0.36) |
| LYS | 8.27(0.03) | 28.11(0.58) | 10.81(0.90) | 0.24(0.02) | 6.51(0.41) | 0.88(0.03) | 0.86(0.05) | 2.28(0.07) | 1.31(0.05) | 37.27(0.99) | 4.32(0.27) |
| PQG | 8.28(0.02) | 27.08(0.81) | 6.91(0.72) | 0.24(0.01) | 8.69(0.32) | 0.94(0.06) | 0.73(0.04) | 1.94(0.15) | 1.16(0.07) | 38.02(0.91) | 5.56(0.33) |
| WLS | 8.22(0.03) | 28.30(0.64) | 7.25(0.58) | 0.21(0.01) | 8.63(0.29) | 0.87(0.03) | 0.82(0.03) | 1.45(0.08) | 1.22(0.05) | 35.69(0.66) | 6.68(0.58) |
| HLS | 8.22(0.04) | 27.42((0.42) | 6.57(0.55) | 0.22(0.02) | 7.89(0.39) | 0.89(0.05) | 0.89(0.06) | 1.59(0.12) | 1.34(0.06) | 37.64(0.67) | 6.49(0.49) |
| LS | 8.33(0.02) | 27.75(0.47) | 5.46(0.38) | 0.22(0.01) | 5.87(0.17) | 0.86(0.03) | 0.69(0.04) | 1.34(0.10) | 1.30(0.07) | 34.44(0.99) | 11.10(0.40) |
| MH | 8.34(0.03) | 31.37(0.97) | 5.55(0.43) | 0.23(0.02) | 5.70(0.31) | 1.16(0.06) | 0.75(0.03) | 1.52(0.13) | 1.32(0.04) | 34.23(0.98) | 6.47(0.75) |
| XQL | 8.38(0.06) | 26.35(0.44) | 5.80(0.38) | 0.23(0.02) | 7.93(0.38) | 1.09(0.09) | 0.68(0.03) | 1.63(0.27) | 1.33(0.05) | 38.84(0.88) | 6.58(0.95) |
| BTM | 7.87(0.05) | 35.29(0.98) | 4.23(0.34) | 0.23(0.02) | 2.79(0.21) | 0.73(0.06) | 0.61(0.05) | 0.86(0.05) | 1.70(0.13) | 36.81(0.66) | 9.68(0.74) |
| SWD | 7.99(0.11) | 29.57(1.34) | 6.94(0.52) | 0.17(0.02) | 3.01(0.31) | 0.58(0.04) | 0.72(0.06) | 0.72(0.08) | 1.47(0.07) | 40.77(0.64) | 8.48(0.50) |
| DHY | 7.27(0.10) | 20.54(1.04) | 5.37(0.53) | 0.11(0.01) | 6.00(0.73) | 0.98(0.08) | 1.06(0.23) | 0.55(0.03) | 1.48(0.05) | 46.07(1.90) | 8.33(0.90) |
| SNJ | 7.90(0.12) | 32.93(2.13) | 2.54(0.14) | 0.14(0.01) | 3.39(0.62) | 0.76(0.09) | 0.60(0.05) | 0.51(0.06) | 2.03(0.07) | 36.15(1.02) | 12.02(0.63) |
| HH | 6.96(0.10) | 17.53(0.88) | 11.77(1.37) | 0.09(0.01) | 3.36（0.49） | 0.80(0.09) | 4.15(1.04) | 0.45(0.06) | 1.55(0.09) | 42.61(2.29) | 4.29(0.60) |
| MLZ | 7.49(0.06) | 29.60(1.08) | 7.96(0.91) | 0.12(0.02) | 1.22（0.12） | 1.62(0.20) | 1.87(0.48) | 0.41(0.05) | 1.67(0.09) | 40.47(0.67) | 8.53(0.64) |
| HPS | 8.40(0.03) | 23.57(0.47) | 2.90(0.26) | 0.15(0.01) | 4.98（0.17） | 1.95(0.14) | 0.89(0.04) | 0.81(0.04) | 2.20(0.09) | 36.97(0.51) | 9.48(0.32) |
| BDGS | 7.64(0.08) | 31.77(1.14) | 7.07(0.37) | 0.24(0.03) | 1.34（0.14） | 1.61(0.23) | 1.77(0.35) | 0.62(0.06) | 1.19(0.08) | 40.64(1.41) | 5.68(0.39) |
| BYS | 7.46(0.06) | 36.49(1.29) | 4.89(0.36) | 0.21(0.01) | 1.22（0.14） | 2.64(0.16) | 2.24(0.26) | 0.37(0.03) | 1.71(0.11) | 33.40(1.11) | 6.79(0.29) |
| GWJ | 7.57(0.11) | 31.99(1.08) | 7.90(0.60) | 0.26(0.04) | 2.51（0.33） | 1.25(0.14) | 1.15(0.27) | 0.97(0.18) | 1.49(0.10) | 37.60(1.26) | 7.94(0.73) |
| FJS | 7.91(0.03) | 35.85(0.98) | 4.89(0.29) | 0.30(0.02) | 1.30（0.08） | 1.56(0.07) | 1.09(0.05) | 0.81(0.05) | 1.40(0.05) | 38.17(0.64) | 6.01(0.33) |
| HS | 7.83(0.06) | 35.05(0.73) | 6.23(0.44) | 0.33(0.03) | 2.09（0.17） | 1.94(0.14) | 1.34(0.06) | 0.95(0.07) | 1.67(0.09) | 33.31(0.78) | 9.02(0.54) |
| MES | 7.93(0.03) | 39.77(0.93) | 4.04(0.28) | 0.30(0.02) | 1.21（0.13） | 1.50(0.11) | 0.83(0.05) | 0.51(0.10) | 1.88(0.12) | 37.79(0.71) | 4.60(0.12) |
| HP | 8.02(0.02) | 36.07(0.87) | 5.40(0.24) | 0.32(0.02) | 1.63（0.16） | 2.49(0.24) | 0.99(0.02) | 0.59(0.07) | 2.05(0.09) | 37.50(0.51) | 5.19(0.15) |
| DYS | 7.79(0.07) | 35.70(2.35) | 5.44(0.75) | 0.37(0.03) | 1.60（0.23） | 0.60(0.10) | 0.97(0.13) | 0.48(0.12) | 1.96(0.14) | 39.80(1.17) | 4.65(0.23) |
| DMS | 7.34(0.07) | 42.83(1.88) | 6.04(0.67) | 0.33(0.04) | 1.19（0.10） | 0.11(0.03) | 0.73(0.07) | 0.27(0.13) | 2.56(0.15) | 33.22(1.01) | 4.90 (0.22) |

Table S3 Pearson correlation coefficients and principal components analysis scores of sum signal intensity for key functional gene categories (n=240)

|  | Fthfs | FBPase | rubisco | cellobiase | endoglucanase | chitinase | mannanase | xylanase | Phenol oxidase | amyA | urec | narg | nirs/k | nosz | nifh | phytase | ppx | ppk | PC1 | PC2 |
| --- | --- | --- | --- | --- | --- | --- | --- | --- | --- | --- | --- | --- | --- | --- | --- | --- | --- | --- | --- | --- |
| Fthfs | 1 | -0.28** | -0.20** | 0.22** | -0.19** | -0.32** | -0.39 | -0.42** | 0.29** | -0.25** | 0.60** | 0.13 | 0.11 | 0.31** | -0.02 | 0.29** | 0.19** | -0.24** | -0.58 | 0.16 |
| FBPase | -0.28** | 1 | 0.09 | -0.49** | 0.26** | 0.06 | 0.21** | 0.65** | -0.30** | -0.14* | -0.33** | -0.20** | -0.33** | -0.60** | -0.13* | -0.41** | 0.24** | 0.11 | 0.58 | -0.65 |
| Rubisco | -0.20** | 0.09 | 1 | -0.01 | 0.15* | 0.14* | 0.15* | 0.13* | -0.26** | 0.12 | -0.17** | -0.12 | 0.16* | -0.02 | 0.11 | -0.36** | -0.04 | 0.27 | 0.34 | 0.28 |
| Cellobiase | 0.22** | -0.49** | -0.01 | 1 | -0.12 | -0.09 | -0.13* | -0.30** | 0.34** | 0.18** | 0.28** | 0.10 | 0.17* | 0.27** | -0.21** | 0.22** | -0.34** | -0.02 | -0.39 | 0.46 |
| Endoglucase | -0.19** | 0.26** | 0.15* | -0.12 | 1 | -0.01 | 0.39** | 0.38** | -0.55** | 0.43** | -0.02 | -0.01 | -0.05 | -0.31** | -0.05 | -0.75** | 0.25** | 0.75** | 0.69 | 0.27 |
| Chitinase | -.32** | 0.06 | 0.14* | -0.09 | -0.01 | 1 | 0.24** | 0.21** | -0.20** | 0.23** | -0.49** | -0.01 | -0.13* | 0.11 | 0.19** | -0.17** | -0.30** | 0.22** | 0.34 | 0.10 |
| Mannanase | -0.39** | 0.21** | 0.15* | -0.13* | 0.39** | 0.24** | 1 | 0.52** | -0.37** | 0.38** | -0.39** | -0.35** | 0.00 | -0.27** | -0.02 | -0.42 | -0.12 | 0.38** | 0.66 | 0.07 |
| Xylanase | -0.42** | 0.65** | 0.13* | -0.30** | 0.38** | 0.21** | 0.52** | 1 | -0.37** | 0.20** | -0.52** | -0.32** | -0.35** | -0.48** | -0.08 | -0.47** | -0.08 | 0.29** | 0.75 | -0.40 |
| Phenol oxidase | 0.29** | -0.30** | -0.26** | 0.34** | -0.55** | -0.20** | -0.37** | -0.37** | 1 | -0.42** | 0.08 | 0.34** | -0.03 | 0.31** | -0.32** | 0.72** | -0.14* | -0.55** | -0.75 | -0.27 |
| amyA | -0.25** | -0.14* | 0.12 | 0.18** | 0.43** | 0.23** | 0.38** | 0.20** | -0.42** | 1 | -0.12 | -0.04 | -0.04 | -0.13* | 0.11 | -0.40** | -0.26** | 0.34** | 0.45 | 0.47 |
| ureC | 0.60** | -0.33** | -0.17** | 0.28** | -0.02 | -0.49** | -0.39** | -0.52** | 0.08 | -0.12 | 1 | 0.08 | 0.06 | 0.04 | -0.08 | 0.17** | 0.16* | -0.07 | -0.47 | 0.27 |
| narG | 0.13 | -0.20** | -0.12 | 0.10 | -0.01 | -0.01 | -0.35** | -0.32** | 0.34** | -0.04 | 0.08 | 1 | -0.08 | 0.12 | -0.18** | 0.31** | 0.07 | -0.20** | -0.38 | -0.06 |
| nirS/nirK | 0.11 | -0.33** | 0.16* | 0.17* | -0.05 | -0.13* | 0.00 | -0.35** | -0.03 | -0.04 | 0.06 | -0.08 | 1 | 0.28** | 0.20** | -0.07 | 0.15* | 0.03 | -0..16 | 0.53 |
| nosZ | 0.31** | -0.60** | -0.02 | 0.27** | -0.31** | 0.11 | -0.27** | -0.48** | 0.31** | -0.13* | 0.04 | 0.12 | 0.28** | 1 | 0.19** | 0.37** | 0.04 | 0-.09 | -0.51 | 0.46 |
| nifH | -0.02 | -0.13* | 0.11 | -0.21** | -0.05 | 0.19** | -0.02 | -0.08 | -0.32** | 0.11 | -0.08 | -0.18** | 0.20** | 0.19 | 1 | -0.14* | -.08 | 0.18** | 0.13 | 0.39 |
| phytase | 0.29** | -0.41** | -0.36** | 0.22** | -0.75** | -0.17** | -0.42** | -0.47** | 0.72** | -0.40** | 0.17** | 0.31** | -0.07 | 0.37** | -0.14* | 1 | -0.14* | -.70** | -0.83 | -0.27 |
| ppx | 0.19** | 0.24** | -0.04 | -0.34** | 0.25** | -0.30** | -0.12 | -0.08 | -0.14* | -0.26** | 0.16* | 0.07 | 0.15* | 0.04 | -0.08 | -0.14* | 1 | -0.05 | 0.71 | -0.15 |
| ppk | -0.24** | 0.11 | 0.27** | -0.02 | 0.75** | 0.22** | 0.38** | 0.29** | -0.55** | 0.34** | -0.07 | -0.20** | 0.03 | -0.09 | 0.18** | -0.70** | 0-.05 | 1 | 0.66 | 0.48 |

Asterisks represent significance of Pearson relationship (*, *P* <0.05; **, *P* <0.01 ).

| **Table.S4 Pearson correlation coefficients between soil chemistry variables and plant Shannon-Weaver index in the study plots (n=240)**   | Environmental factors | Soil pH | Soil  moisture | Total  nitrogen | Total  phosphorus | Soil organic  carbon | Ammonium  nitrogen | Nitrate  nitrogen | Available  nitrogen | Available  phosphorus | Plant Shannon  Weaver index | | --- | --- | --- | --- | --- | --- | --- | --- | --- | --- | --- | | Soil pH | 1.00 | -0.57^**^ | -0.33^**^ | 0.05 | -0.37^**^ | -0.51^**^ | -0.26^**^ | -0.53^**^ | 0.43^**^ | -0.60^**^ | | Soil moisture | -0.57^**^ | 1.00 | 0.59^**^ | 0.31^**^ | 0.58^**^ | 0.62^**^ | 0.29^**^ | 0.73^**^ | -0.34^**^ | 0.48^**^ | | Total nitrogen | -0.33^**^ | 0.59^**^ | 1.00 | 0.47^**^ | 0.95^**^ | 0.74^**^ | 0.30^**^ | 0.86^**^ | -0.06 | 0.25^**^ | | Total phosphorus | 0.05 | 0.31^**^ | 0.47^**^ | 1.00 | 0.29^**^ | 0.19^**^ | 0.35^**^ | 0.50^**^ | 0.00 | 0.08 | | Soil organic carbon | -0.37^**^ | 0.58^**^ | 0.95^**^ | 0.29^**^ | 1.00 | 0.83^**^ | 0.17^**^ | 0.78^**^ | -0.01 | 0.20^**^ | | Ammonium nitrogen | -0.51^**^ | 0.62^**^ | 0.74^**^ | 0.19^**^ | 0.83^**^ | 1.00 | -0.04 | 0.67^**^ | -0.04 | 0.25^**^ | | Nitrate nitrogen | -0.26^**^ | .029^**^ | 0.30^**^ | 0.35^**^ | 0.17^**^ | -0.04 | 1.00 | 0.49^**^ | -0.07 | 0.29^**^ | | Available nitrogen | -0.53^**^ | 0.73^**^ | 0.86^**^ | 0.49^**^ | 0.78^**^ | 0.67^**^ | 0.49^**^ | 1.00 | -0.19^**^ | 0.41^**^ | | Available phosphorus | 0.43^**^ | -0.34^**^ | -0.06 | .00 | -0.01 | -0.04 | -0.07 | -0.19^**^ | 1.00 | -0.45^**^ | | Plant Shannon-  Weaver index | -0.60^**^ | 0.48^**^ | 0.25^**^ | 0.08 | 0.20^**^ | 0.25^**^ | 0.29^**^ | 0.41^**^ | -0.45^**^ | 1.00 |   Asterisks represent significance of Pearson correlation (** *P* < 0.01; * *P* < 0.05). |
| --- | --- | --- | --- | --- | --- | --- | --- | --- | --- | --- | --- | --- | --- | --- | --- | --- | --- | --- | --- | --- | --- | --- | --- | --- | --- | --- | --- | --- | --- | --- | --- | --- | --- | --- | --- | --- | --- | --- | --- | --- | --- | --- | --- | --- | --- | --- | --- | --- | --- | --- | --- | --- | --- | --- | --- | --- | --- | --- | --- | --- | --- | --- | --- | --- | --- | --- | --- | --- | --- | --- | --- | --- | --- | --- | --- | --- | --- | --- | --- | --- | --- | --- | --- | --- | --- | --- | --- | --- | --- | --- | --- | --- | --- | --- | --- | --- | --- | --- | --- | --- | --- | --- | --- | --- | --- | --- | --- | --- | --- | --- | --- | --- | --- | --- | --- | --- | --- | --- | --- | --- | --- |


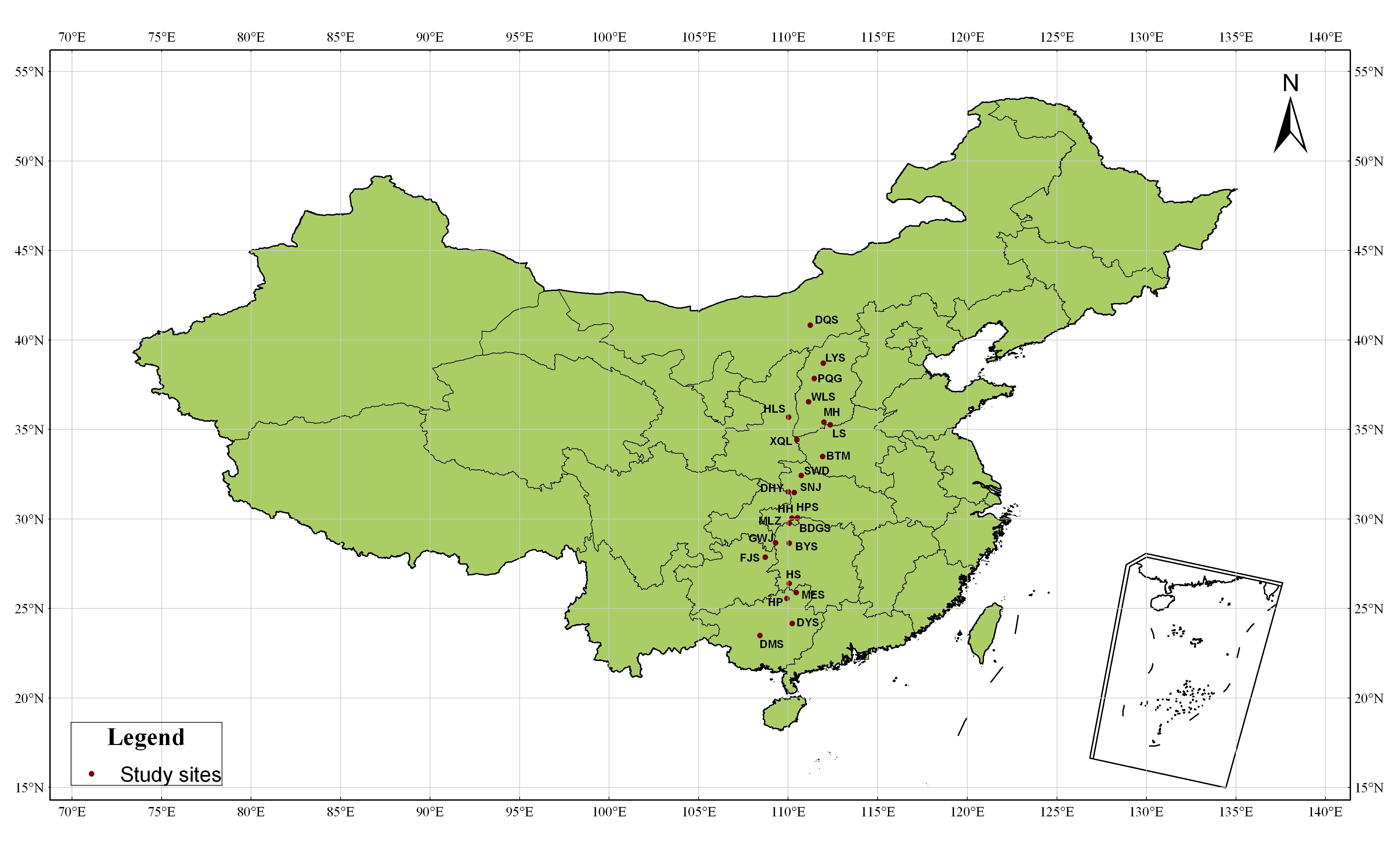


Figure S1. Sampling site map. The map was generated using the vector data by ARCGIS 10.3 software ([http://www.arcgis.com](http://www.arcgis.com/)). DQS, Daqingshan national nature reserve; LYS, Luyashan national nature reserve; PQG, Pangquangou national nature reserve; WLS, Wulushan national nature reserve; HLS, Huanglongshan national nature reserve; LS, Lishan national nature reserve; MH, Manghe national nature reserve; XQL, Xiaoqinling national nature reserve; BTM, Baotianman national nature reserve; SWD, Saiwudang national nature reserve; DHY, Duheyuan national nature reserve; SNJ, Shennongjia national nature reserve; HH, Houhe national nature reserve; MLZ, Mulinzi national nature reserve; HPS, Hupingshan national nature reserve; BDGS, Badagongshan national nature reserve; BYS, Baiyunshan national nature reserve; GWJ, Gaowangjie national nature reserve; FJS, Fanjingshan national nature reserve; HS, Huangshan national nature reserve; MES, Maoershan national nature reserve; HP, Huaping national nature reserve; DYS, Dayaoshan national nature reserve; DMS, Damingshan national nature reserve.

Figure S2. The principal component analysis of the soil chemistry variables.
